# Trends Towards Enhanced Rates and Sex Parity in HPV Vaccination in Croatia (2016–2023)

**DOI:** 10.3390/vaccines13040410

**Published:** 2025-04-15

**Authors:** Lucija Raic, Ivana Pavic Simetin, Emanuel Bradasevic, Antea Jezidzic, Tatjana Nemeth Blazic, Tamara Poljicanin

**Affiliations:** 1 Division for Health Informatics and Biostatistics, Croatian Institute of Public Health, 10000 Zagreb, Croatia; emanuel.bradasevic@hzjz.hr (E.B.); antea.jezidzic@hzjz.hr (A.J.); 2 Division for School Medicine, Mental Health and Addiction Prevention, Croatian Institute of Public Health, 10000 Zagreb, Croatia; ivana.pavic@hzjz.hr; 3 Department for HIV, Sexually Transmitted and Blood-Borne Infections, Croatian Institute of Public Health, 10000 Zagreb, Croatia; tatjana.nemeth-blazic@hzjz.hr; 4 Zagreb County Health Center, 10430 Samobor, Croatia; tamara.poljicanin@gmail.com

**Keywords:** human papillomavirus, vaccination, reproductive health

## Abstract

Background/Objectives: Human papillomavirus (HPV) is a recognized cause of cervical cancer and is associated with several other malignancies, including those affecting the vagina, vulva, anus, penis, and head and neck. The introduction of the HPV vaccine has enabled the prevention of HPV-related cancers. This study aimed to determine the HPV vaccination coverage and examine trends in HPV vaccination in Croatia from 2016 to 2023 in the context of the national vaccination program. Methods: This retrospective study analyzed the aggregated school doctors’ data from 2016 to 2023. HPV vaccination coverages within the 2000–2008 birth cohorts were assessed based on the number of doses administrated, sex, and vaccination schedule, while for the trend analysis joinpoint regression was used. The vaccination coverage between sexes was tested using the chi-square test for trends and their ratio was calculated. Results: The HPV full-dose vaccination coverage increased significantly among the observed birth cohorts, from 4.49% in 2000 to 36.88% in 2008, with an APC = 33.97 and 95% CI: 29.37–42.43 (females from 7.74% to 44.98%, males from 1.44% to 29.14%). The highest recorded vaccination coverage was in the one-dose category (2008 female—52.78%). The vaccination coverage of females was significantly higher than that of males (the chi-square for the linear trend = 659.59, *p* < 0.001) and the female–male ratio decreased from 5.39 in 2000 to 1.54 in 2008. Conclusions: In Croatia, HPV vaccination coverage has increased since the introduction of the national HPV vaccination program. This positive trend was present in both sexes, and the rate ratio between female and male cohorts decreased.

## 1. Introduction

Human papillomavirus (HPV) infection is the main cause of cervical cancer and is related to carcinogenesis in other malignant diseases, such as vaginal, vulvar, anal, penile, and head and neck cancer [1]. HPV is also responsible for other diseases, such as recurrent juvenile respiratory papillomatosis and genital warts [2]. Globally, 0.8% of cancer in men and 8.6% of cancers in women are attributable to HPV [3]. Cervical cancer is the fourth most frequent cancer among women worldwide, and the second most common female cancer in women aged 15 to 44 years worldwide, as well as in the European Union. Each year, there are around 33,000 cases of cervical cancer in the EU and 15,000 deaths due to cervical cancer [4,5].

In recent years in Croatia, we have seen a downward trend in the standardized incidence rate of cervical cancer, while mortality is stable. According to the latest published data, 276 women were diagnosed with cervical cancer in Croatia in 2020 (standardized rate 12.6/100,000 inhabitants), and a third of them were under the age of 50. Additionally, 590 women were diagnosed with carcinoma in situ of the cervix, and a third of them were under the age of 40. Unlike most other types of cancer, cervical cancer occurs at a slightly younger age and is the third most common type of cancer among women aged 30 to 39 years [6,7]. There is a total of 1.80 million women 15 years and older who are at risk of becoming infected with HPV in Croatia [8].

Due to the possibility of the prevention of cervical cancer, and the great burden it represents, in November 2020, the World Health Organization adopted the Global Strategy to Accelerate the Elimination of Cervical Cancer (incidence rate < 4/100,000 women). One of the prerequisites for achieving this goal is a high level of vaccination, i.e., it is expected that at least 90% of all girls under the age of 15 will be vaccinated against HPV by 2030 [9]. Similarly, one of the objectives of Europe’s Beating Cancer Plan is to vaccinate at least 90% of the EU target population of girls and to significantly increase HPV vaccination coverage among boys by 2030 [10].

Since 2007, the HPV vaccine has been available in Croatia, and in 2016, the national vaccination program targeting 14-year-old girls and boys was initiated. Vaccination within the national program is delivered through the Service for School and Adolescent Health, covered by health insurance, and voluntary. It is recommended for students in the eighth grade of primary school (14 years old ± 1 year) [11,12]. A catch-up vaccination program is also available, targeting older generations who did not have a chance to be vaccinated before. Catch-up vaccination is free of charge up to and including the age of 25. Also, it is possible to be vaccinated at ages ≥ 9 at parents’ request [12,13].

To date, the published data on HPV vaccination in Croatia include the total number of doses administered in several academic years (2017/2018, 2018/2019, 2019/2020, 2020/2021, 2021/2022, and 2022/2023) and the one-dose vaccination coverage only for the academic year 2022/2023. However, systematic data on HPV vaccination coverage are lacking, which is crucial for monitoring the progress of prevention and evaluating vaccination programs [14,15,16].

The objectives of this study are to calculate the HPV vaccination coverage of the 2000–2008 birth cohorts in Croatia (as they were the primary target of the national vaccination program from 2016 to 2023) to explore trends, specifically by investigating whether vaccination rates are declining or increasing across the observed birth cohorts and assessing the potential differences in the vaccination coverage between sexes.

## 2. Materials and Methods

### 2.1. Data Collection and Categorization

This retrospective study analyses aggregated data from school medicine doctors in Croatia to assess HPV vaccination trends among individuals born between 2000 and 2008. The dataset, made available to the Croatian Institute of Public Health, was compiled from 2016 to 2023. The available data include the participant’s sex and birth year, the number of administrated doses, and whether the vaccination was initiated before or after the age of 16. Demographic information regarding the target birth cohorts (2000–2008), specifically the size of the population and sex distribution, was obtained from the National Bureau of Statistics (DZS).

The quality of the data is contingent on the accuracy and completeness of the routine medical records maintained by school doctors. Although the dataset is considered reliable, variations in record-keeping practices and potential missing information could influence the precision of the findings. The aggregated nature of the data ensures privacy and confidentiality, but it also limits the ability to examine individual-level factors that may impact vaccination trends.

Based on the HPV vaccination guidelines [12] issued by the Ministry of Health, recommended by the Croatian Society for School and University Medicine of the Croatian Medical Association [17], the Croatian Institute of Public Health, as well as the Centers for Disease Control Prevention [18], we defined different categories and subcategories of vaccination coverage (Table 1). Routine vaccination of the 8th-grade primary school population (14 ± 1 old girls and boys) is recommended, and that population is the primary target of the national vaccination program. If vaccinated between 11 and 15 years, two doses of vaccine are administrated. Thus, the category named “regularly vaccinated“ includes everyone vaccinated ≤15 years with 2 doses. Additionally, catch-up vaccination is available for anyone aged 16–25 who missed regular vaccination. The three-dose schedule applies to persons initiating vaccination at ages 15 through 25 years. Consequently, the “catch-up vaccinated“ category includes everyone vaccinated >15 years with 3 doses. Both regularly vaccinated and catch-up vaccinated categories are considered fully vaccinated and together form a “full dose“ category. According to the most recent recommendations on the use of HPV vaccines issued by the WHO Strategic Advisory Group of Experts (SAGE) on Immunization at its meeting in April 2022, and subsequently endorsed by WHO, national vaccination programs can use either a single-dose or a 2-dose vaccination schedule [19]. Given those recommendations, one-dose HPV vaccination rates were also calculated for all vaccinated with at least one dose. That category is named “one dose“ and includes everybody vaccinated with at least one dose, regardless of age at the moment of vaccination.

### 2.2. Data Analysis

Based on available data, vaccination coverage was analyzed by each birth year. To examine trends in HPV vaccination, HPV full-dose vaccination coverage per birth year was calculated, as well as HPV one-dose vaccination coverage. Vaccination coverage of regularly vaccinated and catch-up vaccinated, subcategories of full dose, were computed as well. Furthermore, vaccination coverages for all categories and subcategories were assessed by sex. Joinpoint statistical software for analysis of continuous linear trends with change points, i.e., joinpoint, version 5.0 [20], was used to reveal a statistically significant increase in vaccination rates among the birth cohort and to identify significant changes in trends [21]. This method identifies statistically significant changes in trends and estimates the Annual Percent Change (APC) for each detected segment. The dependent variable was the full-dose vaccination rate per 100,000 population, calculated from the total number of vaccinated individuals in each birth cohort. The final model tested for a minimum of zero and a maximum of two joinpoints, and model selection was based on statistical criteria. The APC and corresponding 95% confidence intervals (CIs) were reported to illustrate the significance of the trends observed. OpenEpi-Dose Response: Chi-Square for Trend tool [22] was used to compare vaccination rates between females and males in birth cohorts.

Ethical approval for this research was granted by the CIPH Ethical Committee.

## 3. Results

In total, the HPV vaccination status of 344 439 children was examined, and 48.55% were females. The HPV vaccination coverage increased among the 2000–2008 birth cohorts; 4.49% of the initial 2000 birth cohort was fully vaccinated, and 6.44% were vaccinated with at least one dose. Those rates rose to 36.88% fully vaccinated and 44.22% vaccinated with at least one dose for the last observed birth cohort of 2008 (Table 2).

**Figure 1:**
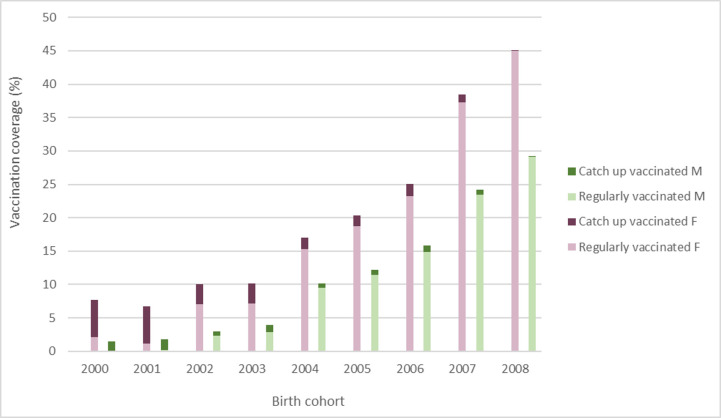
Full-dose coverage shown by sex (F and M) and vaccination schedule (regularly vaccinated and catch-up vaccinated) in 2000–2008 birth cohorts.

To examine trends in the vaccination coverage, the fully vaccinated population was further assessed based on the sex and vaccination schedule (Figure 1). The female vaccination rate increased from 7.74% in the incipient birth cohort to 44.98% in the final birth cohort. An increase in the vaccination coverage was also present in the male population, from 1.44% to 29.14%. The vaccination coverage of females was significantly higher than males in all birth cohorts (extended Mantel–Haenszel chi-square for linear trend = 65.59, *p* < 0.001).

**Figure 2:**
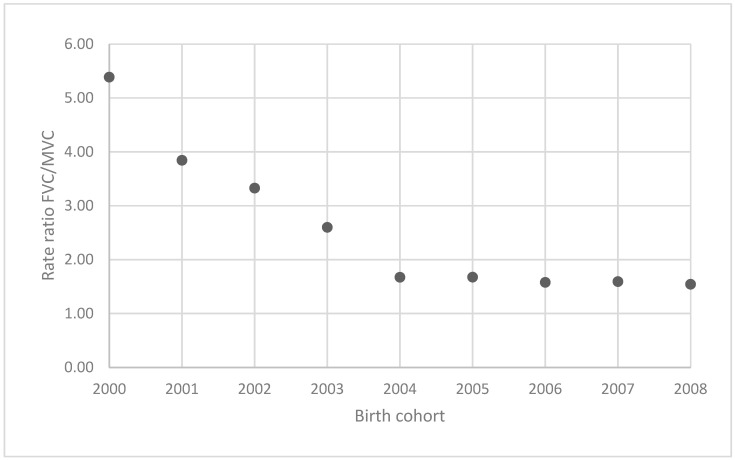
Female vaccination coverage/male vaccination coverage in 2000–2008 birth cohorts.

Furthermore, the ratio of female vaccination coverage (FVC) to male vaccination coverage (MVC) was calculated for each birth cohort (Figure 2). The highest ratio was observed in the initial 2000 birth cohort (5.39) and the lowest in the last 2008 birth cohort (1.54).

**Figure 3:**
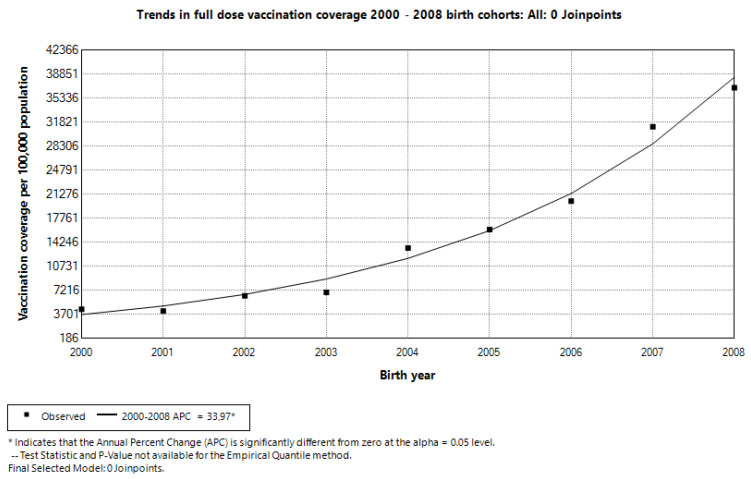
Trends in full-dose vaccination coverage in 2000–2008 birth cohorts.

The joinpoint regression analysis revealed a consistently increasing trend in the full-dose vaccination coverage across the birth cohorts from 2000 to 2008. The final selected model contained zero joinpoints, indicating a single continuous trend without significant changes in the slope (Figure 3). The Annual Percent Change (APC) was estimated at 33.97% (95% CI: 29.37–42.43), demonstrating a statistically significant rise in the vaccination coverage. This suggests that each successive birth cohort experienced an approximate 33.97% annual increase in full-dose vaccination coverage, highlighting a sustained improvement over the study period.

## 4. Discussion

Since the beginning of the national vaccination program in 2016, we have observed an increase in the vaccination coverage among the birth cohorts for all observed categories and subcategories, except the “catch-up vaccinated” category. In the first years (2000 and 2001 birth cohorts) of implementing the national vaccination program, catch-up vaccination was prioritized. In the following years, the number of catch-ups declined because most interested girls and boys were vaccinated through the regular program.

The highest vaccination coverage in all birth cohorts and both sexes was recorded in those vaccinated with at least one dose. As in the rest of the world [19], the response to the first dose is higher than to the additional doses. Current evidence suggests that a single dose of the HPV vaccine offers protection against HPV infection comparable to that of a multi-dose regimen. Accordingly, the World Health Organization’s (WHO) Strategic Advisory Group of Experts (SAGE) on Immunization has supported the optimization of HPV vaccination schedules. As a result, some countries, such as Australia, the United Kingdom of Great Britain, and Estonia, have already moved to single-dose HPV vaccination programs [23].

Although Croatia’s national vaccination program has targeted both sexes from its inception, the vaccination coverage among females has consistently been higher than among males across all birth cohorts. However, this gap has been narrowing over time, with the female-to-male vaccination rate ratio decreasing from 5.4 in the 2000 birth cohort to 1.53 in the 2008 birth cohort. In a broader context, the full-dose vaccination coverage among males in 2023 was estimated at 6% globally, 16% in the European region, and 29.14% in Croatia (2008 birth cohort) [23]. This suggests that Croatia has achieved a relatively high male vaccination coverage compared to other European countries, mainly in the eastern and southeastern regions. For example, male vaccination uptake in 2023 was 15.8% in France, 23.9% in Slovenia, 10% in Slovakia, and only 2.99% in Serbia [24]. Unlike many countries that initially focused only on vaccinating females, Croatia’s program was gender neutral from the outset, which may have contributed to the comparatively higher uptake among males. By now, most European nations have expanded their HPV vaccination programs to include males, and in many of these countries, despite the later inclusion, the female-to-male vaccination ratio is now close to one, reflecting a strong acceptance of gender-neutral vaccination [25].

It is noteworthy that the increase in HPV vaccination rates continued throughout 2020 and 2021 (BC 2005 and 2006) despite the coronavirus pandemic, especially since there was a decline in vaccination rates for other vaccines in the mandatory vaccination program in 2021 in Croatia [15]. The sustained increase in the HPV vaccination coverage in Croatia can be attributed to a combination of factors, the most prominent being the proactive approach of school medicine specialists alongside various promotional activities [26,27]. In Croatia, the vaccination of the school-aged population is facilitated by school doctors who employ a school-based approach to ensure widespread coverage. The school-based approach has been effective in increasing the vaccine uptake by providing onsite vaccination events and improving access to vaccinations [28]. School doctors are encouraged to provide advice about HPV vaccination from grades 5 to 8 of primary school, while an online application for vaccination is introduced in some regions [17]. This school-based approach, reliant on school medicine specialists, is somewhat unique in the European context, where vaccination programs tend to be more fragmented or reliant on family physicians or clinics. This strategy leverages the existing healthcare infrastructure to facilitate vaccine delivery, potentially enhancing accessibility and acceptance among students and parents. Such an approach aligns with practices in countries like Belgium, the United Kingdom, and Nordic countries where school-based vaccination programs have achieved high coverage rates [29,30]. In contrast, countries with low vaccine uptakes, such as Poland (16%), Bulgaria (10%), Slovakia (8%), and Romania (6%), do not have a school-based approach. The low vaccine uptake in those countries can also be attributed to the late introduction of national vaccination programs, weaker public health infrastructure, limited funding, and higher vaccine hesitancy [24,31]. Misinformation about vaccine safety, religious or cultural beliefs, and a lack of strong governmental campaigns, as well as a lack of trust in public health institutions and health authorities, have further contributed to low acceptance rates. Implementing targeted public health initiatives, expanding access through school-based programs, and addressing vaccine misconceptions through community engagement and education campaigns could potentially improve the vaccination coverage in these regions [32,33].

Although an increasing trend in the HPV vaccination in Croatia was present, there is still a gap between the latest vaccination coverage (one-dose 2008 female birth cohort—52.78%) and the WHO’s goals for eliminating HPV (90%). A survey on the self-reported HPV vaccination status among emerging adults in Croatia revealed that 18.3% of participants (25.0% of women and 11.7% of men) reported being vaccinated against HPV, while 21.9% were uncertain of their vaccination status. Among those who were unvaccinated, 35.4% expressed a willingness to receive the vaccine. Vaccination hesitancy was notably lower among women, individuals who perceived a higher risk of STIs, and those who acknowledged the link between HPV and cervical cancer, whereas it was more prevalent among participants with stronger religious beliefs [11]. Possible reasons for HPV vaccine refusal seem to be doubts about the reliability of the vaccine, an uncertainty about vaccine effectiveness, and the fear of a linkage between HPV vaccination and risky sexual behavior [34]. Parental education seems to be one of the possible strategies to develop a positive attitude towards HPV vaccination and could possibly further increase the HPV vaccination coverage [35]. To address these challenges, initiatives such as “Budi mRAK” [26] have been launched to raise awareness about HPV vaccination, combat misconceptions, and reduce vaccine hesitancy. “Budi mRAK” uses various digital platforms to reach the target audience, including social media, websites, and online tools to provide information about the HPV vaccine, its benefits, and the importance of vaccination. Additionally, interactive workshops are being conducted in schools to engage students directly. This initiative also targets parents through digital newsletters, ensuring they receive accurate information and are equipped to make informed decisions about vaccinating their children.

### 4.1. Lessons Learned

The Croatian experience offers several lessons for other countries seeking to enhance their HPV vaccination coverage. The school-based vaccination approach stands out as a best practice. By providing vaccination services directly within schools, Croatia has ensured that vaccines are accessible to a large number of children and adolescents, reducing barriers related to access and convenience. Additionally, the integration of digital tools, such as online applications for vaccination, has streamlined the process and made it easier for families to access the vaccine. A proactive engagement from school medicine specialists has been crucial in Croatia’s ability to sustain the vaccination coverage growth even during the COVID-19 pandemic, demonstrating the importance of maintaining a focus on regular vaccinations despite external disruptions. Furthermore, implementing educational promotional activities through digital platforms can be beneficial in addressing vaccine hesitancy and misinformation, which remain challenges in many countries.

### 4.2. Limitations

The data limitations of our research stem from the narrow scope of this study, which covers 7 years from 2016 to 2023. This period encompasses nine birth cohorts from 2000 to 2008, which were the focus of the national vaccination program. Furthermore, real-world data were used for this study, so related limitations should be considered when interpreting the findings and drawing conclusions from the research. Those included the limited availability of clinically relevant structured data elements and the partial extraction of data from unstructured formats [36], as well as data heterogeneity and challenges of data quality management [37]. Establishing a comprehensive vaccination register could enhance the data quality and surveillance by ensuring standardized data collection, reducing inconsistencies, and enabling more detailed analyses of the vaccination coverage, timing, and individual-level determinants [38]. Croatia has already established an electronic vaccination registry, but it currently includes data only from 2022, and efforts to enhance its data quality are still ongoing.

## 5. Conclusions

There was a positive trend in HPV vaccination coverage in Croatia from 2016 to 2023. Vaccination coverage is continuously increasing among the observed birth cohorts. A positive trend was present in both sexes, and the ratio of rates between female and male cohorts decreased, thus reducing the gap in vaccination rates between them. Further research is needed—not only to analyze current trends, but also to predict future dynamics of HPV vaccination and assess their impact on disease control and eradication.

## Figures and Tables

**Table 1:** Vaccination coverage categories and subcategories based on sex, age of recipient, and number of doses.

Categories	Subcategories	Sex	Age of Recipient	Number of Doses
Full dose	Regularly vaccinated (organized)	Both F and M	≤15	2
	Catch-up vaccinated (opportunistic)	Both F and M	16–25	3
One dose	Both	Both F and M	<25	At least 1

**Table 2:** Full-dose and one-dose vaccination coverage (%) in 2000 to 2008 birth cohorts in whole study population (Total), in females (F) and males (M).

Birth		Full Dose			One Dose	
Cohort	Total	F	M	Total	F	M
2000	4.49	7.74	1.44	6.44	10.67	2.47
2001	4.20	6.77	1.76	7.94	12.20	3.90
2002	6.44	10.06	3.02	9.33	14.15	4.78
2003	6.93	10.14	3.90	10.86	15.47	6.50
2004	13.43	16.96	10.15	20.20	25.11	15.65
2005	16.13	20.32	12.14	25.05	30.41	19.96
2006	20.27	25.02	15.87	34.67	41.55	28.29
2007	31.15	38.49	24.18	39.84	48.81	31.31
2008	36.88	44.98	29.14	44.22	52.78	36.05

## Data Availability

The data presented in this study are available on request from the corresponding author due to privacy and institutional restrictions.

## Abbreviations

The following abbreviations are used in this manuscript:

HPV	Human papillomavirus
WHO	World Health Organization
FVC	Female vaccination coverage
MVC	Male vaccination coverage
APC	Annual Percent Change
